# Fiber-Reinforced Polyester Composites with Photoluminescence Sensing Capabilities for UV Degradation Monitoring

**DOI:** 10.3390/polym14173666

**Published:** 2022-09-03

**Authors:** Carmen R. Tubio, Rubén Seoane-Rivero, Santiago Neira, Vanesa Benito, Koldo Gondra Zubieta, Senentxu Lanceros-Mendez

**Affiliations:** 1 BCMaterials, Basque Center for Materials, Applications and Nanostructures, UPV/EHU Science Park, 48940 Leioa, Spain; 2 GAIKER Technology Centre, Basque Research and Technology Alliance (BRTA), Parque Tecnológico de Bizkaia, Edificio 202, 48170 Zamudio, Spain; 3 IKERBASQUE, Basque Foundation for Science, 48009 Bilbao, Spain

**Keywords:** photoluminescent, polyester-based composite, YAG:Ce,Ga, UV degradation

## Abstract

The wide application of fiber-reinforced polymer composite (FRPC) materials has given rise to the problem of their durability and performance over time. These problems are largely associated with their environmental conditions and service procedures, including ultraviolet (UV) irradiation. Here, we propose the production of polyester-based composites with different contents of synthesized Y_3_Al_5_O_12_:Ce^3+^,Ga (YAG:Ce,Ga) particles to provide sensing abilities towards material degradation. In this regard, the composites were subjected to UV radiation exposure, and its influence on the morphological, mechanical, and optical properties of the materials was investigated. Our findings reveal the self-sensing capabilities of the developed FRPC. The results indicate the potential of the system for the development of highly effective coatings allowing to detect and monitor UV degradation in composite materials for demanding applications.

## 1. Introduction

Fiber-reinforced polymer composites (FRPC) mainly include a reinforcing fiber (glass, carbon, etc.) that imparts specific mechanical properties to enhance the properties of the polymeric matrix, and a matrix material (epoxy, urethane acrylate resins, polyester, vinyl ester, or phenol, among others) [1]. The combination of these different components allows to enhance specific physical and mechanical properties, over conventional materials, which make FRPC ideal candidates for a large variety of applications including aerospace [2], bridge industry [3], and marine industry [4], among others. Furthermore, several factors influence the composite performance and mechanical properties, including the properties of the individual components, the relative amount of different phases, the fiber orientation, and the degree of bonding between the polymer matrix and the reinforcements [5]. Consequently, extensive studies to understand the role of these factors on the resultant mechanical properties of composites have been conducted in recent years in order to expand their applications [6,7,8,9]. For many of these applications, problems arise when the surfaces or coatings of these FRPC are exposed to environmental conditions [10], such as humidity, ultraviolet (UV) light [11], or temperature [12]. In particular, these environmental factors affect their chemical and physical properties, resulting in the degradation of the FRPC materials, and thus affect their durability and efficiency.

Extensive efforts have been conducted to improve damage detection and monitoring, as well as in developing self-repairing FRPC systems. For example, strategies for detecting structural alterations have been explored based on including fiber-optic [13], piezoresistive [14,15], and piezoelectric sensors [16]. On the other hand, the concept of self-repair with healing agents and catalysts has allowed to improve efficiency and durability [17,18]. Moreover, increasing industrial demands for high performing coatings led to the need to improve the properties of conventional polymer coatings, the use of protection or coating layers in different applications being increasingly common to prevent environmental degradation, oxidation, or corrosion, for example. 

In this scope, a necessary improvement in the potential applications can be achieved by exploring the use of fluorescence materials as sensing materials. Fluorescence-based sensors have several advantages such as simple operation, fast response, multiple analysis, high sensitivity, and good selectivity [19]. To date, different strategies have been designed for fluorescence sensors to detect damage or degradation under various environmental conditions in hybrid polymer composites. Fluorescent microcapsules have been developed for the detection of microcracks in epoxy composites [20] and fluorescent indicators have been also introduced in epoxy-based coatings for the early detection of steel corrosion [21]. Similarly, a novel aggregation-induced emission luminogen (AIEgen) has been applied in epoxy coatings, in which the visualization of the early stages of metal corrosion can be realized through a fluorescent ‘Turn-On’ phenomenon [22]. Yellow fluorescent protein has also been used as a mechanoresponsive layer at the fiber/resin interface in glass-fiber-reinforced composites, where the protein loses its fluorescence when subjected to mechanical stress [23]. Finally, self-sensing fluorescence polymer composites have been developed for chemical degradation protection and monitoring in epoxy-based composites [24]. Therefore, fluorescence sensors have shown promising results as they are highly responsive to environmental variations including pH, ions, or UV effect. 

In this context, phosphor materials are excellent candidates as photoluminescent (PL) elements because of their intrinsic characteristics [25,26], where yttrium aluminum garnets (Y_3_Al_5_O_12_, YAG) represent one of the most relevant. In particular, cerium-doped yttrium aluminum garnet (YAG:Ce) phosphor is one of the most used materials, due to the excellent thermal conductivity, high mechanical strength, excellent optical properties, and long lifetime, among others [27]. It has been widely used as a yellow phosphor for white light emitting diodes, detectors, and laser host material, among others. Interestingly, several works showed that the PL emission could be tuned by incorporating substitutional ions in YAG:Ce particles, such as Ga^3+^ [28], Gd^3+^ [29], or other trivalent ions. Therefore, those phosphors with good PL properties can be applied for sensing applications.

In this work, we propose a novel approach to fabricate FRPC-based composites in which phosphor particles are used as sensing active materials. We implement this strategy in a simple laminate structure morphology to demonstrate how by combining polyester-based composites and synthesized YAG:Ce,Ga particles in the interlayer region, it is possible to obtain sensing capabilities to monitor degradation under UV exposure. The effect of UV treatment on morphological, mechanical, and optical properties of the composites has been addressed and the correlation between composition and properties discussed, opening a path for developing new protective coatings with sensing capabilities.

## 2. Materials and Methods

### 2.1. Materials

Yttrium (III) nitrate hexahydrate (Y(NO_3_)_3_·6H_2_O, 99.8% trace metals basis, Sigma-Aldrich, Saint Louis, MO, USA), cerium (III) nitrate hexahydrate (Ce(NO_3_)_3_·6H_2_O, 99% trace metals basis, Sigma-Aldrich, Saint Louis, MO, USA), gallium (III) nitrate hydrate (Ga(NO_3_)_3_, 99.9% metal basis, Alfa Aesar), and aluminum nitrate nonahydrate (Al(NO_3_)_3_·9H_2_O, ACS reagent, ≥98%, Sigma-Aldrich, Saint Louis, MO, USA) were used as metal precursors in the preparation of the YAG:Ce,Ga particles. Acetic acid (Sigma-Aldrich, Saint Louis, MO, USA) and ethylene glicol (EG, Fisher Scientific S.L., Madrid, Spain) were selected as complexing and polymerization agents, respectively.

The polyester resin used for the study was DISTITRON 5119 E1SX20Q (Polynt S.p.A., Italy) which is cured by maintaining 24 h at room temperature, using methyl ethyl ketone (MEK) and cobalt octoate (Co Oct.) as catalyst. Fiber glass MAT EM 1002/300/125 (Krosglass S.A., Krosno, Poland) and fiber glass surface tissue MAT veil (Comargo Composites S.L., Zamudio, Spain) were used as reinforcements. All chemicals were used without further purification. The chemical and physical characteristics of the resin and fiber glass are presented in Table 1.

### 2.2. Synthesis of YAG:Ce,Ga Particles

**Figure 1:**
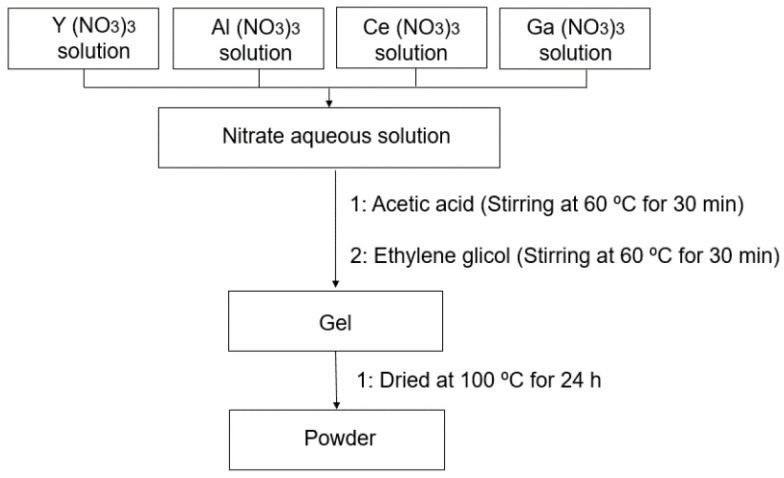
Schematic flow chart outlining the procedure for the YAG:Ce,Ga NPs preparation.

The YAG:Ce,Ga particles were synthesized according to a previously reported sol-gel method [28,30]. In particular, the stoichiometric composition Y_3_(Al_0.8_Ga_0.2_)_5_O_12_:Ce was prepared. A schematic flow chart is shown in Figure 1. Briefly, Y(NO_3_)_3_·6H_2_O (8.43 g, 0.022 mol), Ce(NO_3_)_3_·6H_2_O (0.72 g, 0.002 mol), Ga(NO_3_)_3_ (1.68 g, 0.007 mol), and Al(NO_3_)_3_·9H_2_O (11.28 g, 0.03 mol) were mixed and dissolved in 80 mL of H_2_O under stirring at ambient temperature. Then, acetic acid (1.51 mL) was added to the nitrate aqueous solution, and stirred for 30 min at 60 °C. EG (2.15 mL) was added and stirred for 30 min at 60 °C. Subsequently, the obtained gel was heated at 100 °C for 24 h. Finally, powders were calcined at 600 °C for 3 h with a heating rate of 10 °C/min, and then at 1100 °C for 5 h at a heating rate of 10 °C/min. The obtained powders were analyzed by X-ray diffraction (XRD) measurements using a Philips X’Pert, PRO diffractometer with CuKα radiation (λ = 0.1541 nm). Data were collected in the range of 5–90° (2*ϴ*) with a step size of 0.05°. The morphology and of the powders were studied by scanning electron microscopy (SEM) using Hitachi S-4800 microscope at 20 kV. Before the analysis, samples were sputtered with a 10 nm thin gold layer.

### 2.3. Preparation of the Laminated Composites

**Figure 2:**
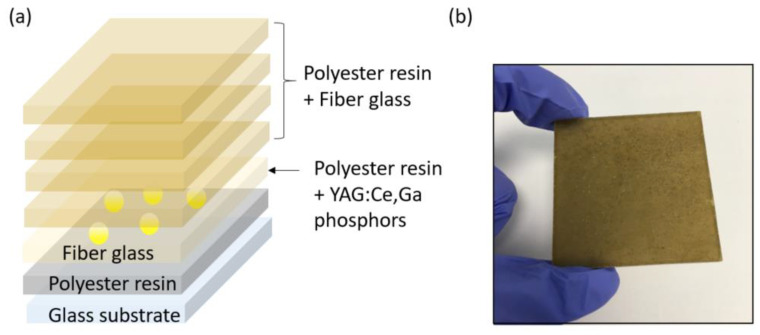
(**a**) Multilayer structure of the laminate composites. (**b**) Optical image of the laminate composites.

The polyester-based composites were prepared using a laminate sequence. The laminate involves several layers (Figure 2a). First, a layer of polyester resin with 0.8% Cobalt Octoate (promoter), 1% methyl ethyl ketone MEK (initiator), and silicone gel was first deposited in the glass substrate. Then, a layer of fiberglass MAT veil was placed on the first layer. This fiberglass was used as reinforcement agent. The functional layer consisting of polyester resin and YAG:Ce,Ga particles (1, 3, and 5 wt.%) was then deposited. Then, four layers of fiberglass MAT with polyester resin were stacked in order to achieve mechanically consistent samples. Finally, the laminate was encapsulated within a plastic film bag. The samples were cured at room temperature under vacuum at 0.98 atm. An example of the laminate samples, with an average thickness of 4 mm, is shown in Figure 2b.

### 2.4. Degradation Tests and Characterization Techniques

The samples were subjected to UV-accelerated aging testing, using an accelerated weathering tester chamber (Q-LAB) with UVB-313 nm lamp. Following the UNE EN ISO 4892-3:2016, method c, cycle 6 standard, the test was carried out within three intervals, including (a) 1000 h of UV irradiation, (b) 8 h of UV radiation at 70 °C without water condensation, and finally (c) 4 h of UV radiation at 50 °C with water condensation.

The morphology of the samples was evaluated by scanning electron microscopy (SEM, Zeis EVO 50) with an accelerating voltage of 20 kV. Energy dispersive X-ray (EDX) analysis was carried out with an INCA detector (Oxford Instruments, Abingdon, UK). All samples were gold-palladium coated using a Leica EM SCD005 sputtering equipment. Further, the surface topography of the samples was analyzed using a 3D optical profiler PLμ NEOX (Sensofar, Barcelona, Spain).

Mechanical properties of the samples were measured with a universal testing machine (Autopraph AG-X 100 kN, Shimadzu Corporation, Japan) and relevant parameters such as Young’s modulus, tensile strength, flexural modulus, and flexural strength were obtained. All the tests were carried out at a speed rate of 2 mm/min and with a load cell of 5 kN. For the flexural measurements, samples of dimensions 15 × 2.9 × 3.8 mm were prepared. In the case of tensile tests, the samples dimensions were 25 × 3 × 3.8 mm Three samples of each composite were tested and the average value was reported. Barcol hardness testing was performed in accordance with UNE-EN 59:2016 test standard specifications using a hardness tester (GYZJ 934-1, Scheider Electric, France).

In order to study the fluorescence response and dispersion of YAG:Ce,Ga particles in the resin before and after UV exposure treatment, a confocal microscope Zeiss LSM 700 with a Axio Observer Z1m was used. This test was carried out using excitation at 488 nm. Steady-state fluorescence measurements were performed with a fluorescence spectrometer FLS980 (Edinburgh Instruments Ltd., Livingston, UK) equipped with a 450 W Xe lamp, double excitation and emission monochromators, and photomultiplier tube detector (RED-PMT) cooled by a Peltier system. The monochromator at 400 nm was used at the excitation and emission arms. The color changes (Δ*E*) in the samples were also investigated using a CM-2300 d spectrophotometer (Konica Minolta), according to the standard UNE EN ISO 11664-4.

## 3. Results and Discussion

### 3.1. YAG:Ce,Ga Particles Characterization

**Figure 3:**
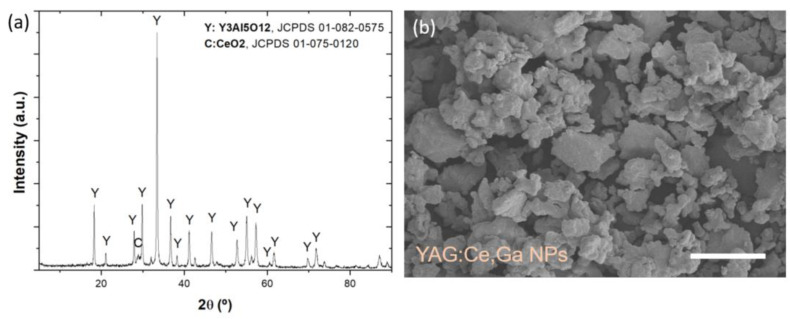
(**a**) XRD pattern of the YAG:Ce,Ga powder calcined at 1100 °C. (**b**) SEM image of the calcined YAG:Ce,Ga powder. Scale bar: 20 µm.

The particles were prepared by sol-gel method, followed by calcination at 1100 °C. As shown in Figure 3a, the XRD pattern of the obtained powders confirms the presence of Y_3_Al_5_O_12_ and CeO_2_ phases, and no impurity peaks appear related to Ga^3+^, in agreement with previous literature [28]. Additionally, Figure 3b shows a representative SEM image of the calcined powders, showing that particle diameter of the powders ranges from 5 to 10 µm. 

### 3.2. Morphological Characterization

**Figure 4:**
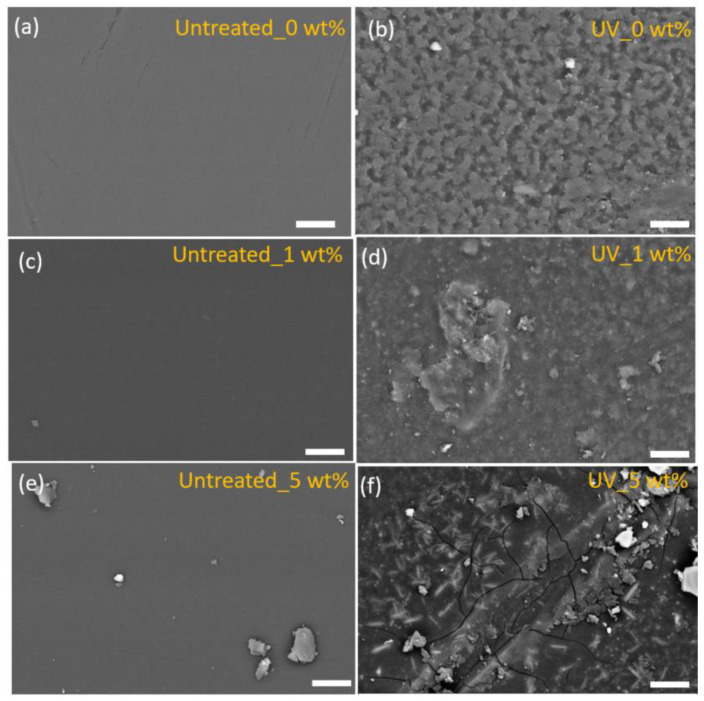
Surface SEM images of untreated samples: (**a**) 0 wt.%, (**c**) 1 wt.%, and (**e**) 5 wt.% NPs, and after UV exposure: (**b**) 0 wt.%, (**d**) 1 wt.%, and (**f**) 5 wt.% NPs. Scale bar: 10 µm.

The representative SEM images obtained from the samples with different YAG:Ce,Ga filler contents (0, 1, and 5 wt.%) before and after UV exposure are shown in Figure 4. Particularly, the untreated samples possess a smooth surface without imperfections. In contrast, the surface of the samples after exposure to photodegradation by long-term exposure to UV light is severely damaged and defects are clearly identified. These results indicate that UV exposure has a strong impact on the surface integrity of the samples. In addition, microcracks appear on the samples with high content of NPs, indicating a possible correlation between the photodegradation and the presence of NPs in the polymer composite. A similar phenomenon has been previously reported in fiber-reinforced composites [31,32]. 

The elemental analysis of the sample surfaces before and after UV treatment was performed by EDX analysis (Table 2). The main elements on the surface of the different samples are C, O, and Si, with high content of C and O, as corresponds to the polymer composition. EDX analysis further shows that the Al and Ce elements are identified in the samples with YAG:Ce,Ga particles after photodegradation. This indicates the degradation of the surface of the laminate composite under the UV treatment. Specifically, the four-layer coating of fiberglass reinforced polyester was completely degraded after UV exposure (Figure 4f).

**Figure 5:**
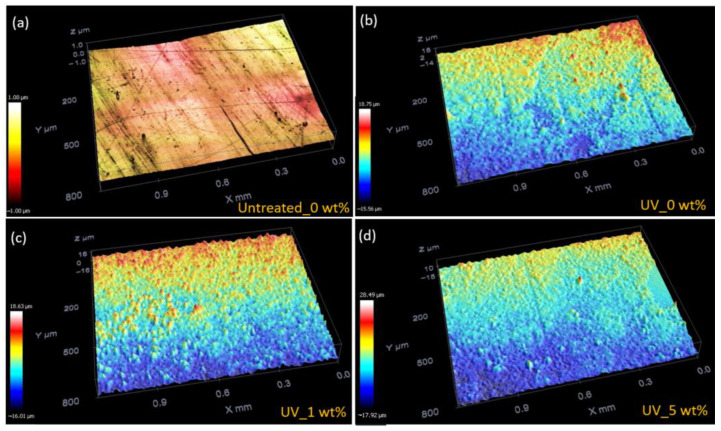
Representative 3D height maps for different samples: (**a**) Untreated samples with 0 wt.% NPs, and UV-treated samples with (**b**) 0 wt.%, (**c**) 1 wt.%, and (**d**) 5 wt.% NP content.

Further, the effect of UV treatment on surface roughness was also investigated (Figure 5a–d) and the mean surface roughness (*Sa*) values before and after the UV treatment are displayed in Table 3. The results show similar values for all untreated samples, *Sa* values of around 12 nm. In contrast, the average surface roughness after UV exposure is much higher when compared to the untreated samples. These results indicate that UV treatment led to significant modifications of the morphology of the samples, the mean surface roughness after UV treatment increasing in the presence of fillers from 78.3 nm (0 wt.%) to 3200 nm (5 wt.%), also confirming the photodegradation of neat polyester with fiber glass.

The photodegradation of the polyester matrix is attributed to absorption of UV radiation and activation of excited states in macromolecules, leading both to the observed chemical and morphological variations [5].

### 3.3. Mechanical Characterization

**Figure 6:**
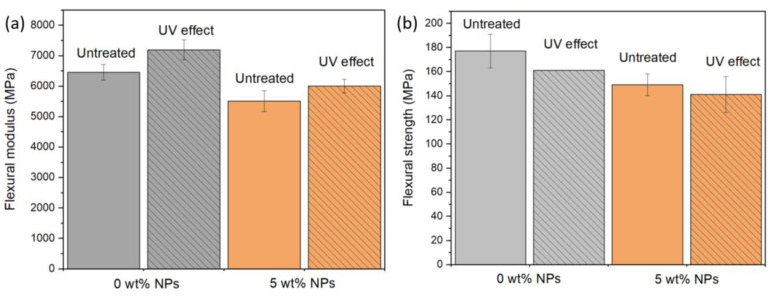
Effect of UV exposure on the (**a**) flexural modulus and (**b**) flexural strength of different samples.

Figure 6a,b shows the comparison between the flexural strength and flexural modulus of representative samples before and after the UV exposure. The results confirm that the flexural properties decrease around 15% with the presence of NPs in the samples. Regarding the effect of UV treatment, the flexural modulus of all samples increases around 8%, while the flexural strength values decrease by around 10%. Overall, findings indicate that the presence of NPs and UV treatment has no significant influence on the flexural properties. This may be attributed to both the fiber-dominated effect on the mechanical response [33] and the fact that photodegradation just affects the top layers of the samples, as discussed previously. 

**Figure 7:**
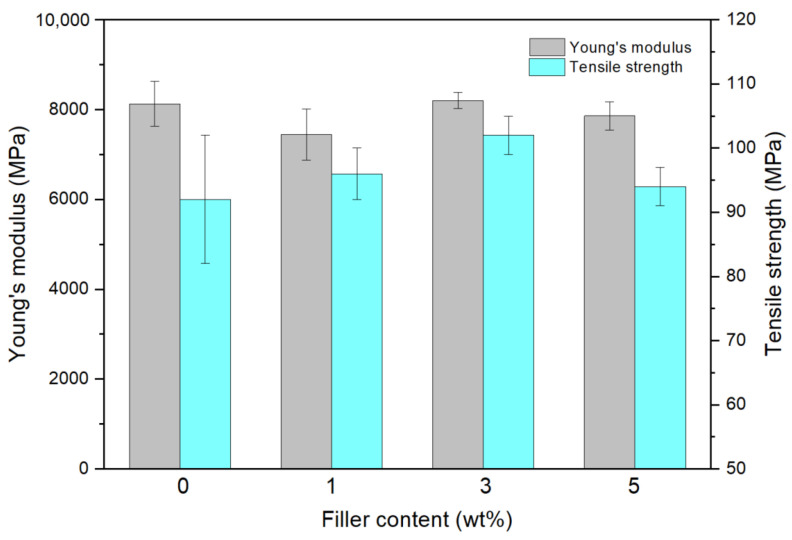
Effect of NP content on the tensile properties of the samples.

Regarding the tensile tests, Figure 7 shows the effect of NPs content on the Young’s modulus and tensile strength of the samples. It is observed that there are no strong variations in these mechanical properties, with maximum values of 8202 ± 179 MPa and 102 ± 3 MPa, respectively at 3 wt.% filler content, as observed in previous studies [24]. Moreover, at 5 wt.% filler content, these values decrease due to filler agglomerations, leading to poor interfacial bonding between polyester matrix and filler [34]. Similar results are obtained after the UV treatment, where no significant influence in the mechanical properties was detected, due to the degradation effect being confined to the surface of the samples. 

Additionally, the Barcol hardness was evaluated, which can be used as an indicator of samples’ surface degradation. The results obtained for the test are provided in Table 4, showing that the effect of UV treatment on the hardness of the samples is around 6.5% for the composite with 0 and 3 wt.% NPs, and around 12–14% for the other samples, which is in agreement with the observed surface modifications of the samples under photodegradation. 

### 3.4. Optical Characteristics

**Figure 8:**
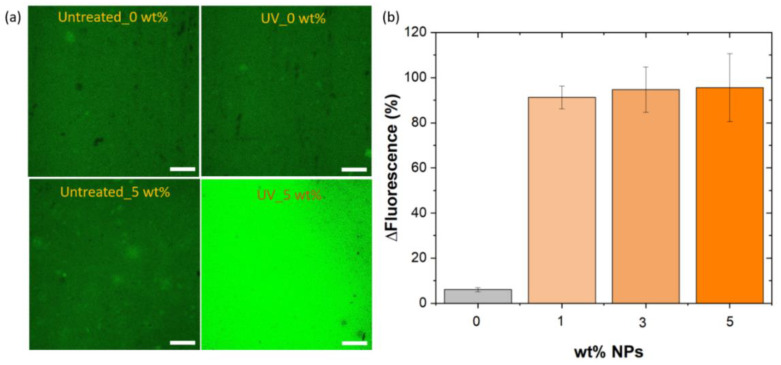
(**a**) CLSM images of the layered composite with 0 and 5 wt.% NP content, before and after the exposure to UV treatment. Scale bar 150 µm. (**b**) Variation of fluorescence detected in the different samples confocal microscopy images taken before and after UV treatment.

In order to demonstrate the potential sensing ability of the composite by employing the YAG:Ce,Ga particles, the luminescence response was investigated by confocal laser scanning microscopy (CLSM). In Figure 8a, we report the CLSM surface images of the samples with 0 and 5 wt.% filler contents, which were obtained before and after the UV exposure. In these images, bright green spots are uniformly dispersed in the matrix, which suggests a good dispersion of particles within the polyester-based matrix. Then, the variation of fluorescence intensity of the samples (Figure 8b) was determined from the confocal images. Significant increase of the intensity was observed after the UV treatment in the samples with NPs, while the sample without NPs has insignificant variation. Therefore, it can be concluded that the change in the fluorescence of the samples after the UV treatment can be used for the evaluation of polymer degradation under UV exposure. Further, the suitability of confocal fluorescence imaging as an imaging tool for monitoring sample degradation was confirmed. 

**Figure 9:**
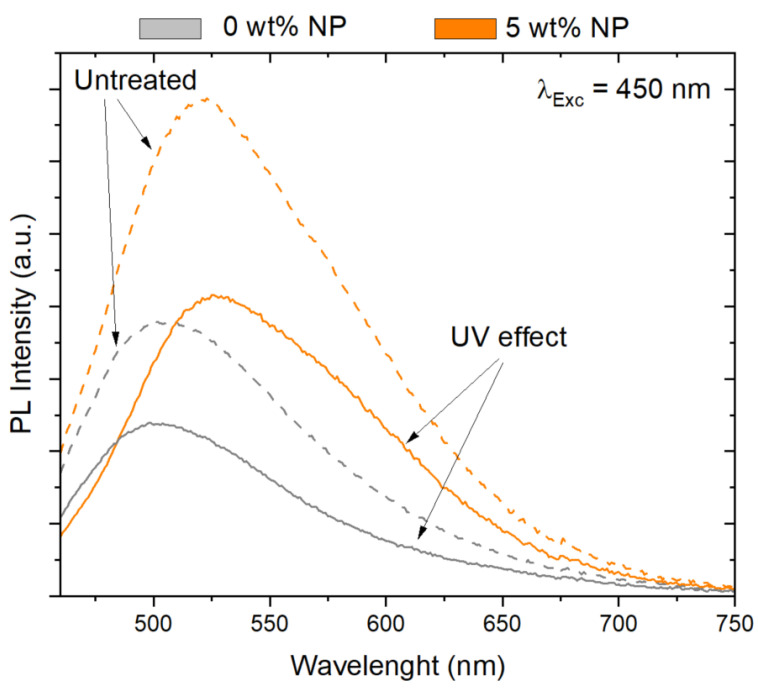
PL spectra of samples with 0 and 5 wt.% NP before (discontinuous line) and after UV treatment (continuous line).

Further experiments using photoluminescent spectroscopy were carried out to evaluate the concentration and UV exposure-dependent variation of the photoluminescence of the samples. Figure 9 depicts the photoluminescence spectra of the composites with 0 and 5 wt.% NPs before and after UV exposure. According to the fluorescence spectra of the untreated samples (discontinuous line), it is worth noting that the emission intensity increases upon filler addition. This phenomenon demonstrates that the particles are well-dispersed in the resin. Additionally, the composite with 5 wt.% NP filler content exhibits an emission peak located at 521 nm. This emission peak matches well with previous studies, where it is found that YAG:Ce NPs synthetized by sol-gel method are characterized by an emission band centered at about 525 nm, and doping with Ga^3+^ changes the spectrum by shifting the peak to lower wavelength centered at 522 nm [21]. At the same time, the spectrum of the composite with particles shows a shift toward longer wavelength. Further, with respect to the fluorescence after the UV treatment (continuous line), the PL intensity was found to decrease up to around 3.5·10^5^ times, indicating a significant PL response of the samples related degradation and changes under UV exposure. It is noted that the fluorescence-dependent variation found in these studies is different compared to those found in the previous confocal studies, which is related to the fact that whereas in confocal just fluorescence that is produced from very close to the focal plane is detected, while fluorescence spectroscopy measurements can be affected by the presence of fiber glass in the first layer of the laminated composites.

**Figure 10:**
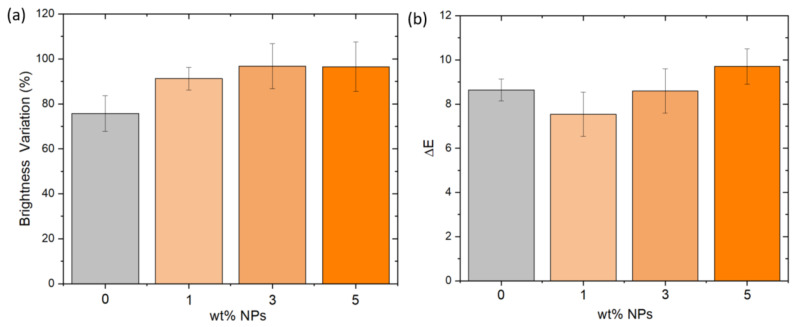
(**a**) Brightness variation and (**b**) color changes of the different samples.

Finally, the factors of brightness and color were investigated. Specifically, Figure 10a,b shows the brightness variation and color changes values (Δ*E*), respectively. The effect of UV radiation is more significant in brightness than in color properties, obtaining up to 90% brightness variation in all samples. Moreover, it is notable that brightness variation increased with filler content, a trend that can be observed in color properties, too. Remarkably, color changes were higher than 5, leading to changes visible by naked eye. These factor-dependent studies provide remarkable evidence of the UV damage effect, consistent with the previous morphological results.

## 4. Conclusions

In this study, it has been experimentally demonstrated that the introduction of YAG:Ce;Ga particles into a fiberglass reinforced polyester-based composites provides sensing capabilities able to detect polymer surface degradation under UV environmental exposure. The YAG:Ce;Ga particles were implemented in a laminated system to demonstrate that by confining the particles in the intermediate layer, it is possible to achieve a sensing response. Morphological characterization revealed that UV treatment significantly changes the surface, roughness, and elemental composition, with the presence of elements of particles for the higher filler contents. On the other hand, the NPs filler content and the UV treatment has no significant effect on flexural and tensile properties, as well as the Barcol hardness. More importantly, the samples exhibited a significant optical response related morphological and structural changes under UV treatment, allowing to detect damage by fluorescence spectroscopy and confocal microscopy. Results demonstrate that confocal microscopy is more suitable to accurately monitor the surface degradation of the samples. The present work confirms the suitability of phosphors particles for practical applications including pipes, tanks, construction surfaces, and many others subjected to potential environmental degradation, showing the way toward high-efficiency self-sensing coatings.

## Figures and Tables

**Table 1:** Chemical and physical characteristics of resin and fiber glass.

Polyester Resin	
Mass density	1.11 g/cm^3^
Elongation at break	3900 MPa
Tensile strength	50 MPa
Viscosity at 25 °C	670–830 mPa·s
Barcol hardness	42
Fiber glass MAT	
Surface weight	300 g/cm^2^
Breaking force	Min. 50 N
Fiber glass MAT surface veil	
Area weight	30 g/m^2^
Tensile strength	≥40 N/50 mm

**Table 2:** EDX results of samples before and after the UV exposure.

		Element Content (wt.%)					
Samples		C	O	Si	Al	Ce	Au
0 wt.%	Untreated	81.44	15.54	0.33	-	-	2.70
	UV	55.2	39.21	1.34	0.34	-	3.81
1 wt.%	Untreated	79.29	18.14	-	-	-	2.54
	UV	52.03	42.56	1.97	0.60	-	2.83
3 wt.%	Untreated	76.66	18.30	0.43	-	-	4.61
	UV	55.66	39.77	3.69	0.88	-	4.46
5 wt.%	Untreated	81.15	13.78	0.24	-	-	4.83
	UV	56.9	21.43	8.66	0.71	2.77	9.52

**Table 3:** Surface roughness values of the samples before and after the UV treatment.

	Surface Roughness *Sa* (nm)	
Sample	Untreated	UV Exposure
0 wt.%	12.6	78.3
1 wt.%	10.99	1896.6
3 wt.%	12.4	2901
5 wt.%	8.7	3200

**Table 4:** Barcol hardness values of the samples before and after the UV treatment.

	Barcol Hardness	
Sample	Untreated	UV Exposure
0 wt.%	46 ± 1	49 ± 2
1 wt.%	42 ± 5	48 ± 2
3 wt.%	46 ± 2	49 ± 2
5 wt.%	42 ± 2	47 ± 2

## Data Availability

Not applicable.
